# Electrochemical skin conductance is associated with peripheral tissue hypoperfusion in septic patients

**DOI:** 10.1186/s40635-025-00813-0

**Published:** 2025-10-15

**Authors:** Jérémie Joffre, Tomas Urbina, Vincent Bonny, Louai Missri, Juliette Bernier, Lisa Raia, Jean-Luc Baudel, Eric Maury, Hafid Ait-Oufella

**Affiliations:** 1 Intensive Care Unit, Saint-Antoine University Hospital, APHP, Sorbonne University, 75012 Paris, France; 2 Centre de Recherche Saint-Antoine, Inserm UMRS-938, Sorbonne University, 75012 Paris, France; 3 Paris Sudden Death Expertise Center, INSERM Unit 970, Paris Cardiovascular Research Center, 75015 Paris, France

**Keywords:** Sepsis, Autonomic nervous system, Mottling, Peripheral perfusion, Capillary refill time, Outcome

## Abstract

**Background:**

Autonomic nervous system (ANS) dysfunction contributes to the pathophysiology of sepsis. However, studies using reliable methods for ANS activity monitoring and evaluating its association with outcomes in sepsis patients are scarce. The Sudoscan^®^ device offers a non-invasive method to evaluate sympathetic function by measuring electrochemical skin conductance (ESC), but its clinical relevance in sepsis remains unclear. This study aimed to assess autonomic sympathetic activity in septic patients using the Sudoscan^®^ technology and explore its relationship with peripheral perfusion and outcomes.

**Methods:**

This prospective, observational, single-center study included 97 consecutive adult ICU septic patients without or with shock. Sudoscan^®^ measurements were performed at admission and serially for 72 h, alongside standard hemodynamic and peripheral perfusion assessments (e.g., knee capillary refill time [CRT], mottling, cardiac output). Associations between ESC ("sudoscore"), clinical parameters, and mortality at day-28 were analyzed.

**Results:**

Of the 97 septic patients included, 37% had shock. Mottling was frequent (53%), and mean knee CRT was 3.3 ± 2.5 s. The mean admission Sudoscore was 31.2 ± 21 µS and was significantly higher in patients with peripheral perfusion abnormalities, such as mottling compared to no mottling (35.7 ± 21 *vs* 28.5 ± 19.5 µS, *P* = 0.04) and prolonged knee CRT > 5 s compared to CRT < 5 s (44.2 ± 25 *vs* 29.6 ± 18.6 µS, *P* = 0.03). Additionally, Sudoscore positively correlated with CRT (*P* = 0.01, *R* = 0.27). There was no difference in Sudoscore between patients receiving vasopressors or not, and between patients receiving sedative drugs or not. Longitudinally, the Sudoscore course was significantly lower over the first 72 h in survivors compared to non-survivors (*P* = 0.04, two-way ANOVA mixed model effect).

**Conclusion:**

Electrochemical skin conductance measured via Sudoscan^®^ may serve as a surrogate marker of autonomic sympathetic hyperactivation during sepsis and is associated with peripheral circulatory impairment. Although admission values were not independently predictive of mortality, elevated and persistently high Sudoscores are associated with death at day 28. Sudoscan^®^ may offer a non-invasive window into sympathetic activity during septic shock and warrants further investigations.

**Supplementary Information:**

The online version contains supplementary material available at 10.1186/s40635-025-00813-0.

## Background

Sepsis remains a leading cause of morbidity and mortality worldwide. Septic shock, its most severe form, affects 10 to 20% of intensive care patients, with mortality rates ranging from 40 to 60% despite advances in resuscitation strategies [1]. The sepsis pathophysiology involves complex interactions among the immune system, coagulation cascade, microvascular endothelium, and autonomic nervous system (ANS), leading to tissue hypoperfusion [2] and ultimately to life-threatening organ failure. Several bedside tools have been developed to evaluate skin tissue hypoperfusion, such as mottling score and capillary refill time (CRT), both being associated with organ failure severity and outcome at admission and after initial resuscitation. However, the mechanisms responsible for decreased skin microvascular blood flow in the context of sepsis remain not fully understood. Cutaneous tissue perfusion is predominantly regulated by the sympathetic nervous system through myogenic control [3, 4] rather than metabolic demand. Indeed, the ANS can influence skin vasomotor tone: vasoconstriction occurs in response to cold exposure or stress, mediated by noradrenaline acting on α-adrenergic receptors in cutaneous vessels [5]. Conversely, vasodilation results from local cholinergic pathway activation in response to heat stress [6], as well as from central regulatory mechanisms. In addition, afferent thermosensory signals are processed in central structures such as the hypothalamus and medullary raphe, modulating sympathetic outflow to the skin [5]. Moreover, heart rate variability (HRV) and posture-induced changes have been shown to correlate with skin microcirculatory patterns, indicating sympathovagal modulation of peripheral perfusion [7]. Although these findings are mainly derived from studies conducted under homeostatic conditions, we speculated that ANS dysregulation may be involved in impaired local microvascular blood flow in sepsis patients.

Characterization of ANS activity, which integrates appropriate sympathetic and parasympathetic balance, remains challenging in critically ill patients. Still, several studies have reported an association between parameters of ANS dysfunction and outcome. For example, analyzing two hours period plethysmogram tracings within the first 24 h of ICU stay, on 540 non-selected patients, Bodenes et al. found that low HRV was associated with poor outcome in the ICU or at Day-28, independently of the admission diagnosis, treatment, and mechanical ventilation [8]. In a prospective sepsis cohort, De Castillo et al. also reported that low HRV is a strong predictor of mortality [9, 10]. In addition, blood pressure variability has also been associated with poor outcomes in emergency ward patients [11].

Nevertheless, the relationship between ANS dysfunction and peripheral tissue hypoperfusion remains unknown. To address this question, we prospectively explored ANS sympathetic activity in septic patients using the Sudoscan^®^ device, which measures electrochemical skin conductance (ESC) in the extremities [12]. This quick (3-min) procedure assesses sympathetic innervation of sweat glands, with ESC values (expressed in microSiemens, µS) serving as a proxy for C-fiber activity. The eccrine sweat glands, which are densely distributed on the palms and soles, are innervated almost exclusively by postganglionic sympathetic cholinergic fibers. Their activity is under direct sympathetic control, and changes in sudomotor function reflect alterations in sympathetic nerve integrity or activity. In the absence of severe neuropathy, it is a proxy for ANS sympathetic activity. Sudoscan^®^ has been extensively validated in the context of chronic diabetic neuropathy and has also shown potential in detecting peripheral autonomic dysfunction in a variety of other clinical conditions [13–17]. Although the Sudoscan^®^ device has not previously been used in critically ill patients, we hypothesized that patients with sepsis or septic shock may exhibit alterations in ESC, reflecting ANS sympathetic activity. Therefore, this study aimed to explore Sudoscan^®^-derived values in a cohort of sepsis and septic shock patients and to investigate their relationship with peripheral perfusion markers and 28-day mortality.

## Patients and methods

### Data collection

This was a single-center, prospective, observational study conducted over one year. All consecutive patients diagnosed with severe infections with or without shock were included. Patients with severe skin disease or burns affecting hand palms were non-inclusion criteria. Data were collected for all patients at admission (within 2 h), 6 h, 12 h, and 24 h, and then once daily for 3 days. Clinical variables included blood pressure, heart rate, urine output, catecholamine dosages, mottling score, and capillary refill time (CRT) measured at the knee. Left ventricular function and cardiac output were assessed by echocardiography (Vivid 7 Dimension’06, GE, Healthcare^®^). Sudoscan^®^ measurement process is short (<3 mins), non-invasive, and standardized, which minimizes technical variability and patient-dependent factors [12]. Due to patients’ bedridden status, the Sudoscore was measured only on the hands. For unconscious patients, hand placement was performed by the physician using gloves. The average value between the left and right sides was recorded as the Sudoscore. Mortality was assessed at day 7 and 28.

### General management

Patients resuscitation was guided by our local protocol, adapted from international guidelines [18]. Standardized treatment included crystalloid volume expansion and, if necessary, norepinephrine, used in a stepwise manner to achieve pre-defined endpoints: mean arterial pressure (MAP) ≥ 65 mmHg and urinary output ≥ 0.5 ml/kg/h. If required, patients were sedated with propofol and/or midazolam, and analgesia was provided with sufentanil. The use of low doses of hydrocortisone (200 mg/day) was considered when there was persistence of high dosage of vasopressor (>0.25 µg/kg/mn) requirements despite adequate fluid resuscitation. At the time of the study, vasopressin was not used in our department.

### Ethical considerations

The observational protocol was approved by the ethical committee, “Comité de Protection des Personnes” (CPP Ile-de-France 4 approval #CPP-2015/64NI). This observational study involved no specific intervention according to the Sudoscan^®^-recorded values and was classified as a Category 3 Interventional Research Involving the Human Person (RIPH3) according to French regulations. In line with the regulatory framework, written informed consent was not required. Instead, all patients and/or their legally authorized representatives received a detailed information letter explaining the purpose and procedures of the study, along with their rights. They were informed that participation was voluntary and could be declined at any time without affecting their medical care. No data were collected for patients or families who refused participation.

### Statistics

Continuous variables are reported as means (±SD), while categorical variables are expressed as percentages. Univariate analyses were conducted to assess associations between patient characteristics at admission regarding the presence or absence of hypoperfusion and 28-day mortality. Continuous variables were compared using Student’s *t*-test or the Mann–Whitney *U* test, as appropriate, and categorical variables were compared using the *χ*^2^ test. To identify independent predictors of 28-day mortality at admission, variables with *P* values < 0.05 in univariate analysis were included in a multivariable logistic regression model, with “Sudoscore” forced into the model. Model fit was evaluated using the Hosmer–Lemeshow test, and discrimination was assessed using the area under the receiver operating characteristic curve (ROC AUC). For the analysis of repeated measurements across multiple time points, we used a two-way ANOVA with mixed-effects modeling to account for both group and time effects. Statistical analyses were conducted using R software (https://www.R-project.org/) and GraphPad Prism version 9.00 (GraphPad Software Inc.^®^) for graphical representations.

## Results

### Characteristics of patients at admission

A total of 97 consecutive adult septic patients without (63%) or with (37%) shock admitted to the ICU were included in this study. Of these, 55 (56.7%) were male, with a mean age of 66 ± 17 years. The most frequent comorbidities were hypertension (47.4%), diabetes mellitus (26.8%), or cirrhosis (7.2%). Based on Sepsis-3 criteria, 61 patients (62.9%) met the definition of sepsis at admission, while 36 patients (37.1%) were diagnosed with septic shock. The most common primary infection sites were the lungs (51.5%) and abdomen (25.8%). Positive blood cultures were identified in 51.5%.

At day 0, 51 patients (52.6%) required endotracheal intubation/mechanical ventilation, and 52 required vasopressor infusion. Peripheral tissue hypoperfusion was frequent at admission, as 44 (45%) patients had mottling, 11 (11.3%) stage 1, 18 (18.7%) stage 2, and 15 (15.4%) stage 3 or more. Mean Knee CRT was 3.3 ± 2.5 s with 16 (16.5%) values exceeding 5 s. The 7-day mortality rate was 19.7% and the 28-day mortality was 26.8%. Table 1 summarizes patients’ characteristics at inclusion.

**Table 1:** Characteristics of included patients at admission

Variable	All patients (*n* = 97)	No hypoperfusion^a^ (*n* = 51)	Hypoperfusion (*n* = 46)	*P*-value (M-W or Chi^2^)
Age (years, mean ± SD)	66 ± 17	60 ± 19	73 ± 12	0.0008
Male (*n*,%)	55 (56.7)	25 (9)	30 (65.2)	0.15
BMI	25.3 ± 6	26 ± 6.3	24 ± 5.4	0.04
Medical history (*n*,%)				
Hypertension	46 (47.4)	20 (39.2)	26 (56.5)	0.11
Diabetes	26 (26.8)	11 (21.6)	15 (32.6)	0.26
Vascular disease	22 (22.7)	9 (17.6)	13 (28.3)	0.23
CKD	12 (12.4)	3 (5.9)	9 (19.6)	0.06
Cirrhosis	7 (7.1(2)	3 (5.9)	4 (8.7)	0.7
Sepsis primary site (*n*,%)				
Lung	50 (51.5)	28 (54.9)	22 (47.8)	0.26
Abdomen	25 (25.8)	10 (19.6)	15 (32.6)	
Urinary tract	17 (17.5)	11 (21.6)	6 (13)	
Endocarditis	6 (6.2)	1 (2)	5 (10.9)	
CNS	3 (3.1)	2 (3.9)	1 (2.2)	
Skin	1 (1)	0 (0)	1 (2.2)	
Unknown	3 (3.1)	2 (3.9)	1 (2.2)	
Positive blood culture	50 (51.5)	24 (47.1)	26 (56.5)	0.41
Septic shock^b^	36 (37.1)	13 (25.5)	23 (50)	0.02
Admission SAPSII	57.3 ± 23.7	48 ± 20	67 ± 24	< 0.0001
Day 7 mortality	19 (19.6)	4 (7.8)	15 (32.6)	0.004
Day 28 mortality	26 (26.8)	8 (15.7)	18 (39.1)	0.01

*SD* standard deviation, *BMI* body mass index, *CKD* chronic kidney disease, *CNS* central nervous system, SAPSII Simplified Acute Physiology Score II, M-W Mann–Whitney. ^a^No mottling and knee CRT < 5 s. ^B^septic shock according Sepsis-3 definition at inclusion

### Sudoscore at admission

**Fig. 1:**
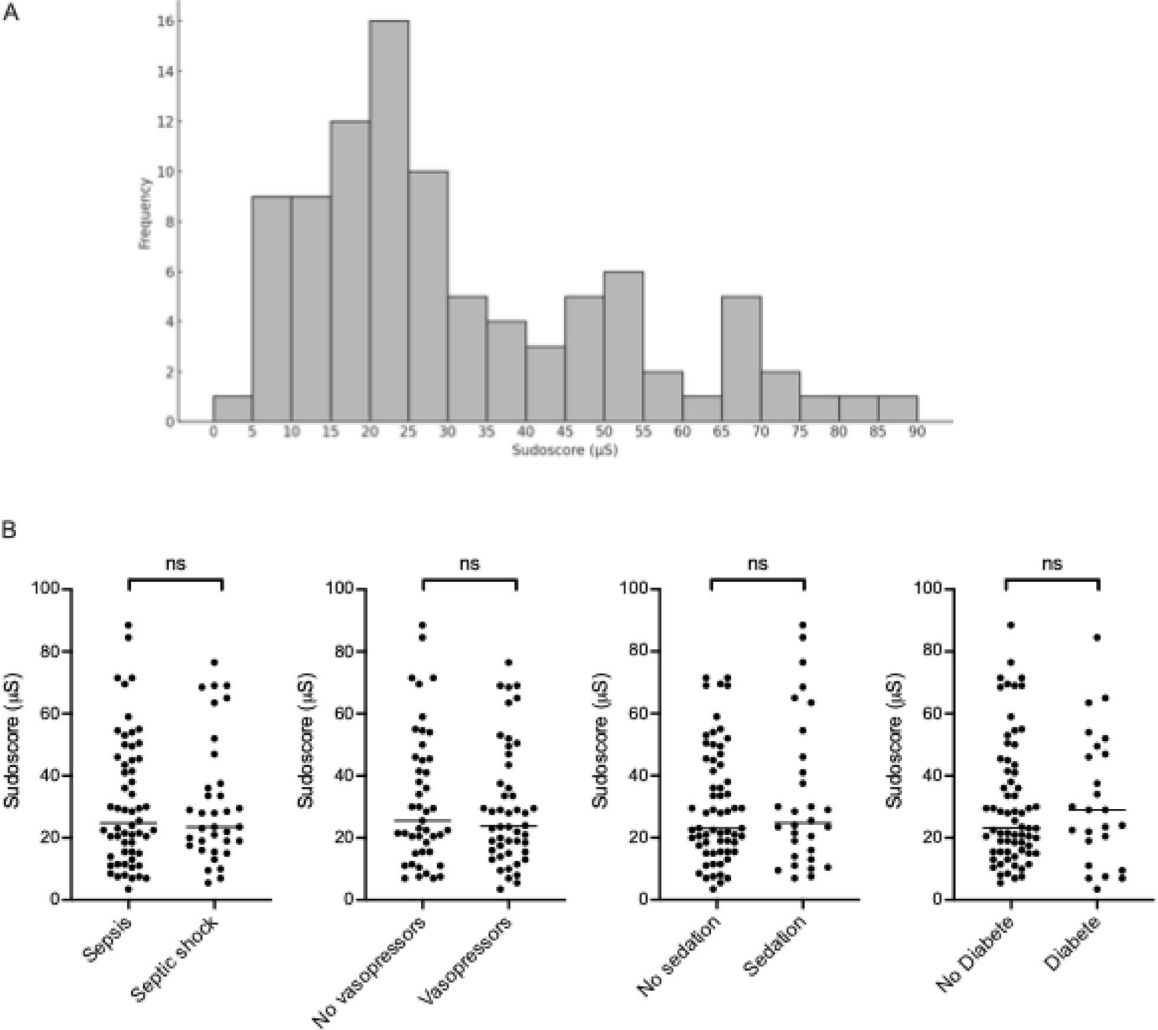
Sudoscore distribution and according to patients’ status. **A** Displays the distribution of hand Sudoscan^®^ values recorded at patients’ inclusion. **B** Shows comparison of Sudoscan^®^ values at inclusion between sepsis and septic shock, patients with or without vasopressors, with or without sedation and with or without medical history of diabetes, respectively

The mean Sudoscore at admission was 31.2 ± 21 µS, values being not normally distributed (Kolmogorov–Smirnov test, *P* < 0.001) (Fig 1A). No significant differences in Sudoscore were observed between septic patients with or without shock, and no difference between those receiving norepinephrine or not. Similarly, Sudoscore values did not differ based on sedation status or diabetes mellitus (Fig 1B).

**Fig. 2:**
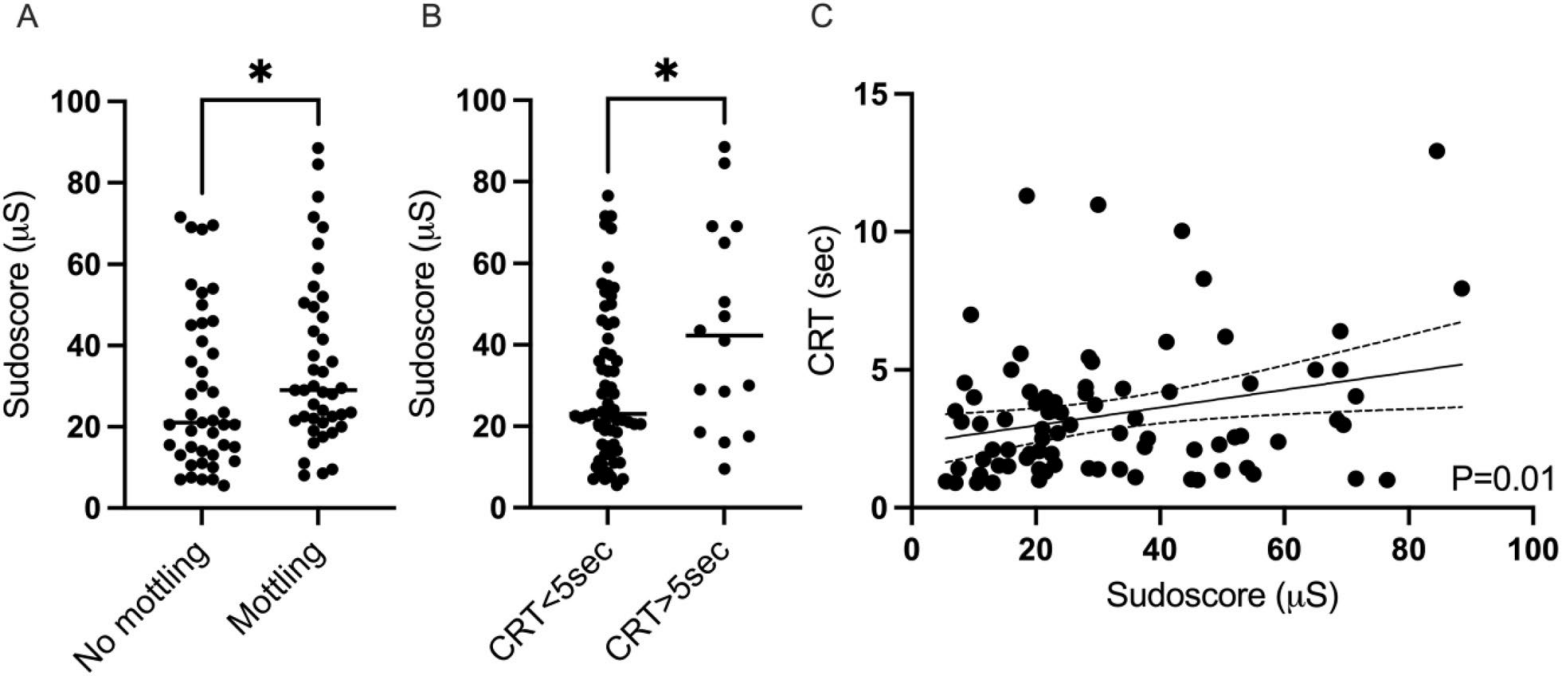
Sudoscore according to peripheral tissue perfusion. Sudoscore according to the presence of mottling (**A**) and Sudoscore in patients with and without prolonged CRT (**B**). Linear correlation between Sudoscore and knee CRT at admission (*P* = 0.014, *R* = 0.27 Pearson correlation coefficient) (**C**). Mottling and CRT were assessed at the knee area *P < 0.05, Mann–Whitney test. *CRT* capillary refill time

Interestingly, Sudoscore was different in patients according to peripheral tissue hypoperfusion assessment. Indeed, Sudoscore was significantly higher in patients with mottling than those without (35.7 ± 21 vs 28.5 ± 19.5 µS, *P* = 0.04) (Fig 2A). Sudoscore was significantly higher in patients with prolonged knee CRT (44.2 ± 25 vs 29.6 ± 18.6 µS, *P*=0.03) (Fig 2B). Finally, the Sudoscore modestly correlates with knee CRT (*P* = 0.014, *R* = 0.27) (Fig 2C); the higher the Sudoscore, the longer the knee CRT.

### Factors associated with 28-day mortality

In a univariate analysis comparing admission parameters between survivors and non-survivors at 28 days, no significant differences were observed between groups in macrocirculatory parameters such as heart rate, mean arterial pressure, or cardiac output. Conversely, the mottling score (1 ± 1.3 vs. 2 ± 1.9; *P* = 0.01), Knee CRT (2.7 ± 2 vs. 4.9 ± 2.9 sec; *P* < 0.001), and lactate levels (2.1 ± 1.1 vs. 5.6 ± 3mmol/L; *P* < 0.001) were significantly lower in the survivors group. The Sudoscore at admission was not different between day-28 survivors and non-survivors (29.9 ± 19.2 vs. 34.9 ± 23.1 µS; *P* = 0.44) (Table 2). To adjust for potential confounding factors, a multivariate analysis was performed, and Sudoscore was still not associated with 28-day mortality. Only admission lactate was significantly associated with 28-day mortality (OR: 2.31 per mmol/L; 95% CI 1.60–3.73; *P* < 0.001) (Supplemental figure 1).

**Table 2:** Univariate analysis of patients’ vitals and in-ICU management variables according D28 survival status

Parameters at admission (mean ± SD)	All patients (*n* = 97)	Survivors (*n* = 71)	Non-survivors (*n* = 26)	*P*-value (M-W or Chi^2^)
MAP (mmHg)	74 ± 12	75 ± 12	71 ± 12	0.07
HR (bpm)	105 ± 27	103 ± 25	109 ± 30	0.39
Core temperature (°C)	37.8 ± 1.5	38 ± 1.5	37.5 ± 1.5	0.24
Mottling score	1.2 ± 1.5	0.91 ± 1.3	1.8 ± 1.9	0.03
CRT Knee (s)	3.3 ± 2.5	2.7 ± 2	4.9 ± 2.9	< 0.0001
Lactate (mmol/L)	3.1 ± 2.4	2.1 ± 1.1	5.6 ± 3	< 0.0001
Cardiac output (L/mn)	4.9 ± 1.9	5 ± 1.9	4.4 ± 1.9	0.26
Sudoscore (mS)	31.2 ± 21	29.9 ± 19.2	34.9 ± 23.1	0.44
Medication/life support				
Mechanical ventilation	38 (39.2)	26 (36.6)	12 (46.2)	0.48
Fluids received before inclusion (mL)	1245 ± 1275	1129 ± 1266	1558 ± 1512	0.18
Antibiotics	98 (100)	71 (100)	26 (100)	> 0.99
Norepinephrine	51 (52.6)	30 (42.3)	21 (80.7)	0.001
Epinephrine	1 (1)	0 (0)	1 (3.9)	0.27
Dobutamine	2 (2)	1 (1.4)	1 (3.9)	0.47
Propofol	14 (14.4)	10 (14.1)	4 (15.4)	0.99
Midazolam	26 (26.8)	15 (21.1)	11 (42.3)	0.07
Sufentanyl	31 (32)	18 (25.3)	13 (50)	0.03
Neuromuscular blockade	9 (9.3)	6 (8.5)	3 (11.5)	0.70

*SD* standard deviation, *MAP* mean arterial pressure, *HR* heart rate, *CRT* capillary refill time, *M-W* Mann–Whitney

**Fig. 3:**
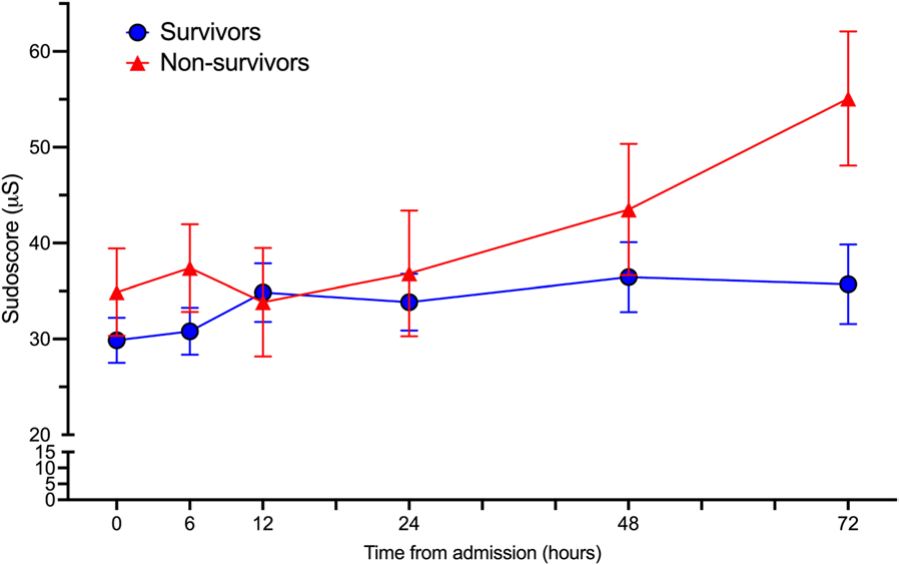
Course of Sudoscore according to the 28-day survival status. Course of Sudoscan^®^ measured ESC values during the first 72 h post-inclusion, comparing survivors and non-survivors at day 28. Two-way ANOVA mixed model (*P* = 0.04 for group and *P* = 0.04 for time)

However, changes in Sudoscore during the first 3 days, evaluated using a two-way ANOVA mixed-effects model, were different according to the outcome. Overall it remains lower in survivors compared to non-survivors (− 7.80 difference; 95%CI [− 15.25 to− 0.35]; *P*=0.04) (Fig 3 and supplemental Table 1). The course of hemodynamic parameters is reported in Supplemental Table 1 and supplemental figure 2.

## Discussion

In this prospective exploratory study of critically ill septic patients, we observed a significant relationship between ANS sympathetic activity estimated by ESC and markers of peripheral tissue hypoperfusion. Furthermore, we observed that persistently high ESC during the first days of ICU stay was associated with 28-day mortality.

Exploring ANS activity is very challenging because such investigation could only be performed with indirect tools that reflect sympathetic–parasympathetic imbalance, such as heart rate or blood pressure variability, which requires monitoring for several hours and additional time for analysis [19]. Here, we used the Sudoscan^®^ technology, which evaluates the ANS sympathetic activity by measuring sweat gland function in only 3 minutes. Its use has primarily been validated in detecting early signs of neuropathy in diabetes. Comparing our Sudoscore values to those reported in the literature, we surprisingly observed that the mean Sudoscore at admission (31.2 ± 21 µS) was significantly lower than values typically described in chronic conditions associated with autonomic neuropathy. For instance, in both healthy subjects and diabetic patients with moderate small-fiber neuropathy, Sudoscore values often exceed 50–60 µS [20] and, in healthy adults, “normal” ESC values range approximately from 60 to 90 μS in the hands and 70 to 90 μS for the feet electrodes, with a slight decline after 70 years. Due to the constraints of bed rest in critically ill patients, we exclusively used the hand plates for Sudoscan^®^ measurements, whereas in ambulatory patients, reported values typically combine data from both hand and foot plates. Therefore, direct comparisons with values from studies in ambulatory populations should be interpreted with caution. Moreover, Sudoscan^®^ has not been specifically used or validated in critically ill patients. Nonetheless, the device operates via a fully automated protocol with minimal operator influence and has demonstrated low intra-individual variability across several clinical populations [12], supporting its measurement robustness and validity in various contexts. Surprisingly, in our cohort, admission Sudoscore values were not significantly different between diabetic and non-diabetic patients. We can speculate that this may be due to all septic patients exhibiting severe ESC impairment as a result of their critical illness or, alternatively, that our diabetic patients did not have neuropathic complications, as none of the non-diabetic patients had clinically apparent neuropathy. We observed a negative relationship between Sudoscore and peripheral tissue perfusion. Sudoscore was higher in mottled patients and those with prolonged knee CRT. In other words, the higher the sympathetic activity, the lower the skin tissue perfusion. Such results could be explained by downstream pathways induced by sympathetic activity, such as adrenergic receptor stimulation promoting vasoconstriction, and ultimately reducing microvascular blood flow and organ perfusion. The significant correlation between knee CRT and Sudoscore supports this hypothesis; however, the weak correlation strength implies that other factors may modulate microvascular blood flow. Indeed, several facets of endothelial dysfunction, previously reported by our group in sepsis patients, are another contribution to vasomotor tone dysregulation and tissue hypoperfusion [21].

As previously reported by our group and others, we found that extended mottling is more frequent and knee CRT is longer in non-survivors [22–24]. As previously reported by our group and others, the CRT cut-off to define prolonged CRT was different according to the site of measurement. The more accurate threshold to predict mortality in the knee area is 5 s, whereas it is lower in the fingertip (2.4 s) [25]. Here, we did not measure fingertip CRT but its accuracy to evaluate severity is similar to knee CRT [25]. Additionally, lactate levels were significantly higher in non-survivors from admission through the resuscitation period. Lactate serves as an integrative marker not only of tissue hypoperfusion, but also of adrenergic stress. In the context of our study, this elevation may indeed reflect persisting autonomic nervous system (ANS) hyperactivity, as suggested by findings from Sudoscan assessments.

In our study, admission sudoscore was not associated with 28-day mortality. It could reflect that all sepsis patients have severe ESC alterations, but also a lack of power of our study as we observed a trend toward a higher sudoscore in non-survivors at day 28. Nevertheless, we observed that Sudoscore changes over time toward increased values were associated with mortality. We can speculate that non-survivor, that persistent elevation in Sudoscore may indicate small-fiber neuropathy secondary to endothelial or/and dysfunction; persistently elevated Sudoscore reflects sustained ANS overactivation and indicates failure to restore homeostasis in response to sepsis. These results align with prior studies in sepsis, showing that ANS dysregulation was associated with increased mortality [9, 26, 27]. Thus, a reduced HRV has been linked to increased severity in sepsis and septic shock, correlating inversely with organ failure scores. Furthermore, favorable clinical outcomes are associated with increasing HRV during resuscitation, whereas persistently low HRV predicts mortality [28, 29]. Therefore, accumulative evidence sets HRV as a key marker of ANS dysfunction, strongly associated with sepsis severity and outcomes in adult and pediatric cohorts [9, 27, 30–34]. This reinforces the idea that ANS activity monitoring at admission and during early resuscitation could be a valuable tool for patient stratification and clinical monitoring.

Our pilot study has several limitations, including its exploratory nature, the lack of validation of the device and reference values in critically ill patients, the lack of continuous monitoring, and the ergonomic constraints of Sudoscan^®^ in the ICU setting. Moreover, no direct comparisons between Sudoscan^®^-derived metrics and HRV were performed. Lastly, as our study began before the 2016 update of the definitions and clinical criteria for diagnosing sepsis, we included patients formerly classified as having severe sepsis and septic shock in order to ensure a broad spectrum of septic patients with varying degrees of severity. Ultimately, the complementary value of Sudoscan^®^ in sepsis and septic shock remains speculative, and further studies incorporating serial measurements and integrating Sudoscan^®^ into a multimodal ANS assessment framework could help refine its role in sepsis prognostication or adjuvant therapy indication.

## Conclusion

In this prospective pilot exploratory study involving septic patients, we found that higher electrochemical skin conductance, measured using the Sudoscan^®^ device as a surrogate for sympathetic autonomic nervous system activity, is associated with clinical markers of peripheral tissue hypoperfusion.

## Supplementary Information

Supplementary file1.

## Data Availability

The dataset analyzed during the current study is available from the corresponding author on reasonable request.
