# Supplementary material for: Electrochemical skin conductance is associated with peripheral tissue hypoperfusion in septic patients
Source: Intensive Care Med Exp. 2025 Oct 15;13:101. doi: 10.1186/s40635-025-00813-0 (PMC12528571; doi:10.1186/s40635-025-00813-0)
Supplement: Supplementary file 1 — Supplementary file1. [file 40635_2025_813_MOESM1_ESM.docx]

**Supplemental figure 1: 28-day mortality predictors multivariate analysis**


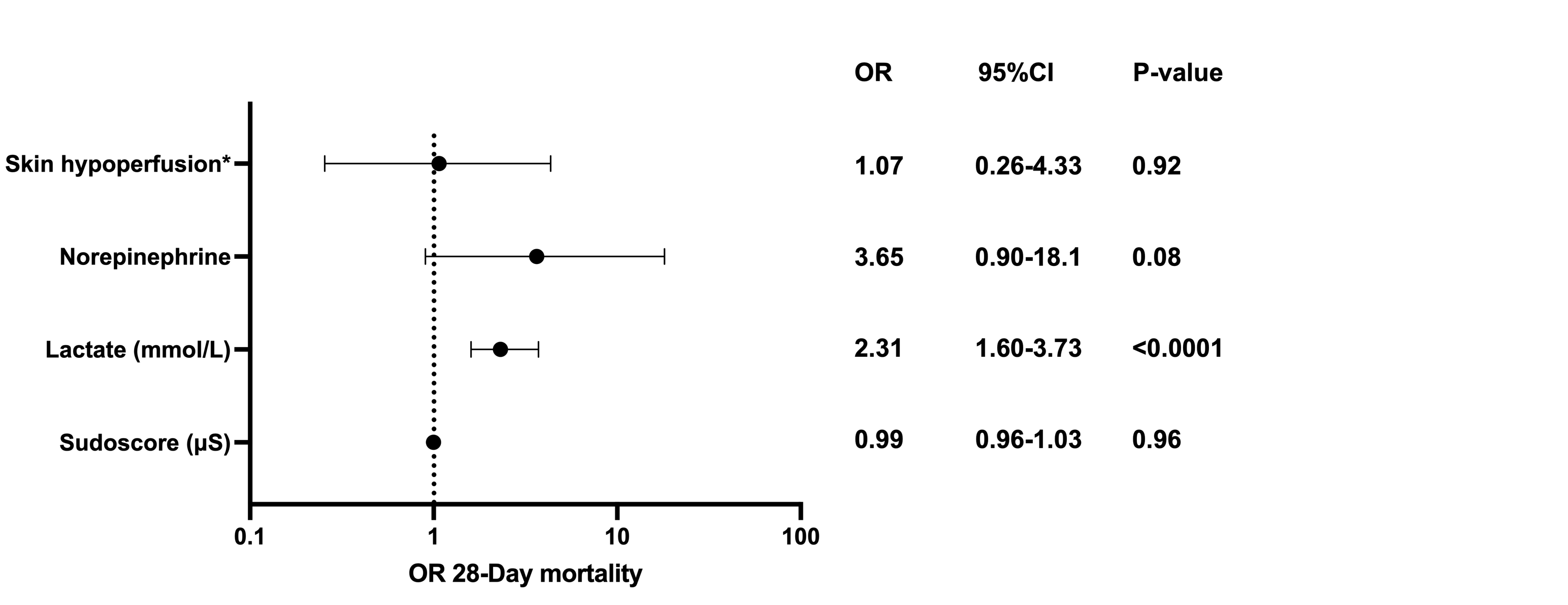


**Supplemental figure 1: legends and abbreviations:** Multivariate analysis (logistic regression, full model) of 28-day mortality risk factors at admission. The dots represent the OR; the line through each dot corresponds to the 95% confidence interval. Goodness of fit (Hosmer-Lemeshow statistic): 10.74 (P=0.22); Turj r2=0.5. Calibration: AUC=0.88 (0.78-0.97); P<0.0001. * Mottling or knee CRT>5sec.

**Supplemental table 1:**

|  |  |  |  |  |  |
| --- | --- | --- | --- | --- | --- |
|  |  | **Survivors**  **(n= 71)** | **Non-survivors**  **(n= 26)** | **P-value** | **Mixed model effect**  **P-value for “group effect”** |
| HR (/min) | Inclusion | 103 ± 25 (n=71) | 109 ± 30 (n=26) | 0.39 | P=0.07 |
|  | H6 | 100 ± 23 (n=65) | 107 ± 28 (n=25) | 0.24 |  |
|  | H12 | 98 ± 22 (n=58) | 102 ± 22 (n=18) | 0.07 |  |
|  | H24 | 96 ± 20 (n=59) | 106 ± 20 (n=10) | 0.06 |  |
|  | H48 | 93 ± 19 (n=42) | 107 ± 24 (n=11) | 0.15 |  |
|  | H72 | 93 ± 32 (n=28) | 90 ± 17 (n=7) | 0.84 |  |
| MAP (mmHg) | Inclusion | 75 ± 12 (n=71) | 71 ± 12 (n=26) | 0.07 | P=0.002 |
|  | H6 | 77 ± 11 (n=64) | 69 ± 11 (n=25) | 0.006 |  |
|  | H12 | 76 ± 10 (n=57) | 68 ± 12 (n=18) | 0.006 |  |
|  | H24 | 77 ± 14 (n=56) | 78 ± 16 (n=13) | 0.82 |  |
|  | H48 | 80 ± 16 (n=41) | 75 ± 16 (n=11) | 0.54 |  |
|  | H72 | 80 ± 11 (n=28) | 76 ± 10 (n=7) | 0.53 |  |
| CO (L/mn) | Inclusion | 5 ± 1.9 (n=67) | 4.4 ± 1.9 (n=23) | 0.26 | P=0.78 |
|  | H6 | 4.7 ± 1.7 (n=48) | 5 ± 3 (n=19) | 0.88 |  |
|  | H12 | 4.8 ± 1.8 (n=42) | 4.6 ± 1.8 (n=15) | 0.96 |  |
|  | H24 | 4.9 ± 1.7 (n=51) | 5 ± 11.2 (n=8) | 0.65 |  |
|  | H48 | 4.7 ± 1.6 (n=30) | 6.1 ± 1.7 (n=7) | 0.08 |  |
|  | H72 | 4.9 ± 1.6 (n=21) | 4.1 ± 1.4 (n=5) | 0.51 |  |
| Sudoscore (µS) | Inclusion | 29.9 ± 19.2 (n=67) | 34.9 ± 23.1 (n=26) | 0.44 | P=0.04 |
|  | H6 | 30.8 ± 19.4 (n=63) | 37.4 ± 21.9 (n=23) | 0.21 |  |
|  | H12 | 35.8 ± 22.8 (n=53) | 32.6 ± 24 (n=19) | 0.49 |  |
|  | H24 | 33.9 ± 21.9 (n=55) | 36.8 ± 22.7 (n=12) | 0.61 |  |
|  | H48 | 36.5 ± 22.5 (n=38) | 43.5 ± 21.7 (n=10) | 0.27 |  |
|  | H72 | 35.7 ± 20.3 (n=24) | 55.1 ± 17 (n=4) | 0.03 |  |
| Mottling score | Inclusion | 0.91 ± 1.3 (n=67) | 1.8 ± 1.9 (n=26) | 0.03 | P<0.0001 |
|  | H6 | 0.82 ± 1.4 (n=62) | 1.6 ± 1.6 (n=17) | 0.02 |  |
|  | H12 | 0.62 ± 1.2 (n=50) | 2.1 ± 1.8 (n=16) | 0.003 |  |
|  | H24 | 0.43 ± 0.98 (n=54) | 1.6 ± 2.2 (n=11) | 0.05 |  |
|  | H48 | 0.56 ± 1.1 (n=36) | 0.7 ± 1.3 (n=10) | 0.83 |  |
|  | H72 | 0.38 ± 0.71(n=24) | 1.1 ± 1.9 (n=7) | 0.33 |  |
| Knee CRT (sec) | Inclusion | 2.7 ± 2 (n=61) | 4.9 ± 2.9 (n=24) | <0.0001 | <0.0001 |
|  | H6 | 2.5 ± 1.6 (n=57) | 5.4 ± 3.8 (n=22) | <0.0001 |  |
|  | H12 | 2.6 ± 1.6 (n=48) | 6.3 ± 5.7 (n=16) | 0.0004 |  |
|  | H24 | 2.3 ± 1.4 (n=53) | 3.2 ± 2.1 (n=11) | 0.37 |  |
|  | H48 | 2.2 ± 1.3 (n=35) | 4.2 ± 3.5 (n=9) | 0.06 |  |
|  | H72 | 2.1 ± 1.4 (n=22) | 4 ± 3.7 (n=6) | 0.2 |  |
| Lactate (mmol/l) | Inclusion | 2.1 ± 1.1 (n=65) | 5.6 ± 3 (n=26) | <0.0001 | P<0.0001 |
|  | H6 | 2 ± 1.1 (n=42) | 5 ± 4 (n=21) | <0.0001 |  |
|  | H12 | 2.1 ± 1.9 (n=38) | 6 ± 5.3 (n=15) | <0.0001 |  |
|  | H24 | 1.8 ± 2 (n=33) | 5.6 ± 6.9 (n=12) | 0.009 |  |

**Supplemental table 1 abbreviations:** MAP, mean arterial pressure; HR, heart rate; CRT, capillary refill time; CO, cardiac output. Tables values are means ± SD.

**Supplemental Figure 2:**


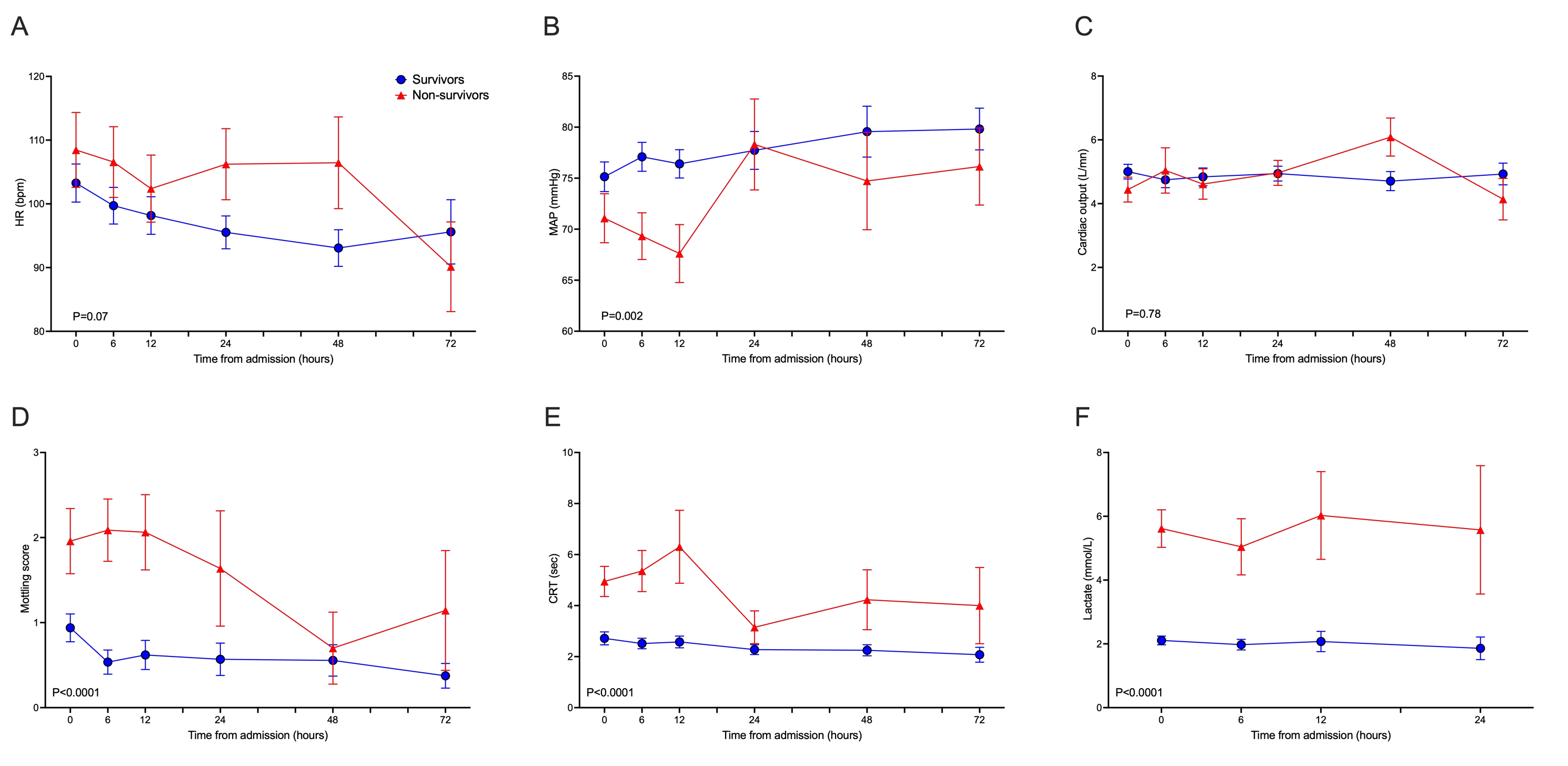


**Supplemental Figure 2 legends and abbreviations:** Evolution of hemodynamics parameters across early resuscitation according to the survivor status at day -28. **A** to **C** report macrohemodynamic (HR, MAP and CO). **D** to **F** report perfusion parameters (mottling score, Knee CRT and arterial lactate). The P-value displayed on the graph is the “group factor” P-value obtained from two-way ANOVA. Abbreviations: MAP, mean arterial pressure; HR, heart rate; CRT, capillary refill time at the knee site; CO, cardiac output.
